# Investigating the causal associations between metabolic biomarkers and the risk of kidney cancer

**DOI:** 10.1038/s42003-024-06114-8

**Published:** 2024-04-01

**Authors:** Lede Lin, Yaxiong Tang, Kang Ning, Xiang Li, Xu Hu

**Affiliations:** 1 Department of Urology and Institute of Urology, West China Hospital, Sichuan University, Chengdu, Sichuan China; 2 Department of Head and Neck Surgery, Sun Yat-sen University Cancer Center, Guangzhou, Guangdong China; 3 State Key Laboratory of Oncology in South China, Guangdong Provincial Clinical Research Center for Cancer, Sun Yat-sen University Cancer Center, Guangzhou, Guangdong China

**Keywords:** Renal cell carcinoma, Genome-wide association studies

## Abstract

Metabolic reprogramming plays an important role in kidney cancer. We aim to investigate the causal effect of 249 metabolic biomarkers on kidney cancer from population-based data. This study extracts data from previous genome wide association studies with large sample size. The primary endpoint is random-effect inverse variance weighted (IVW). After completing 249 times of two-sample Mendelian randomization analysis, those significant metabolites are included for further sensitivity analysis. According to a strict Bonferrion-corrected level (P < 2e-04), we only find two metabolites that are causally associated with renal cancer. They are lactate (OR:3.25, 95% CI: 1.84-5.76, P = 5.08e-05) and phospholipids to total lipids ratio in large LDL (low density lipoprotein) (OR: 0.63, 95% CI: 0.50-0.80, P = 1.39e-04). The results are stable through all the sensitivity analysis. The results emphasize the central role of lactate in kidney tumorigenesis and provide novel insights into possible mechanism how phospholipids could affect kidney tumorigenesis.

## Abstract (web summary)

A multiple Mendelian randomization study emphasizes the central role of lactate in kidney tumorigenesis and provides novel insights into possible mechanism how phospholipids could affect kidney tumorigenesis.

## Introduction

Kidney cancer is increasingly acknowledged as a disease of metabolism^1^. Renal cell carcinoma (RCC), the most common type of kidney cancer, has been identified to hold numerous gene mutations impacting basic metabolic process^2–4^. Those mutations involve in glycolysis, the tricarboxylic acid (TCA) cycle, fatty acid oxidation, glutamine metabolism and so on. However, most of those phenomenon were based on case-control study or sample dataset research and only correlation could be investigated^5,6^. Fortunately, recent publication emphasized the role of several metabolites, including lactate and fatty acid, in tumor growth^7,8^. They drew causal associations between certain metabolites and kidney cancer in cell-based and animal-based models. These publication aroused our reflection that, since quantities of metabolic biomarkers existed in human blood, which ones or even which one played critical roles in kidney cancer occurrence?

As it is impractical to conduct randomized controlled studies (RCTs) to investigate the effect of a certain metabolite concentration on kidney tumorigenesis, we decide to apply Mendelian randomization (MR) analysis to explore the causal relationship between metabolites and kidney cancer, which is thought as the best way to exploring such a question^9,10^. Here, we conducted this multiple two-sample MR study to investigate the causal effect of 249 metabolic biomarkers on kidney cancer from population-based data.

## Results

**Fig. 1:**
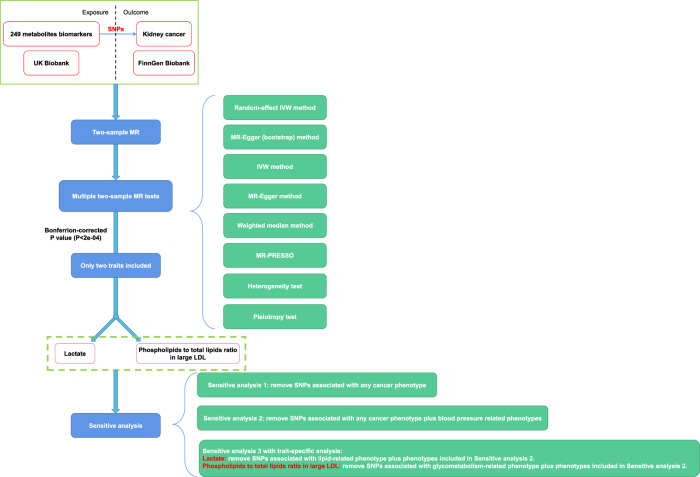
Study flowchart to investigate the causal effect of 249 metabolic biomarkers on kidney cancer. MR Mendelian randomization, IVW inverse variance weighted, MR-PRESSO Mendelian Randomization Pleiotropy RESidual Sum and Outlier, LDL low density lipoprotein, SNPs single nucleotide polymorphisms.

Two hundred and forty-nine metabolites were tested their causal effect on kidney cancer occurrence. Finally, only two metabolic traits were considered causal association with kidney cancer and they went on for sensitivity analysis. The study flowchart was depicted in Fig. 1.

### Multiple two-sample MR analysis between 249 metabolic biomarkers and kidney cancer

Totally, 249 metabolites were conducted two-sample MR analysis on kidney cancer one by one. We summarized several common metabolites’ effect on kidney cancer, including lipidic, glycemic and fatty acid-related traits (Fig. S1). According to two-side *P* value at 0.05 level, we discovered that lactate, sphingomyelins, phosphatidylcholines, linoleic acid, omega-6 fatty acids, omega-3 fatty acids and polyunsaturated fatty acids had suggestive causal association with kidney cancer. All of these metabolites were positively correlated with kidney tumorigenesis (Fig. S1).

**Fig. 2:**
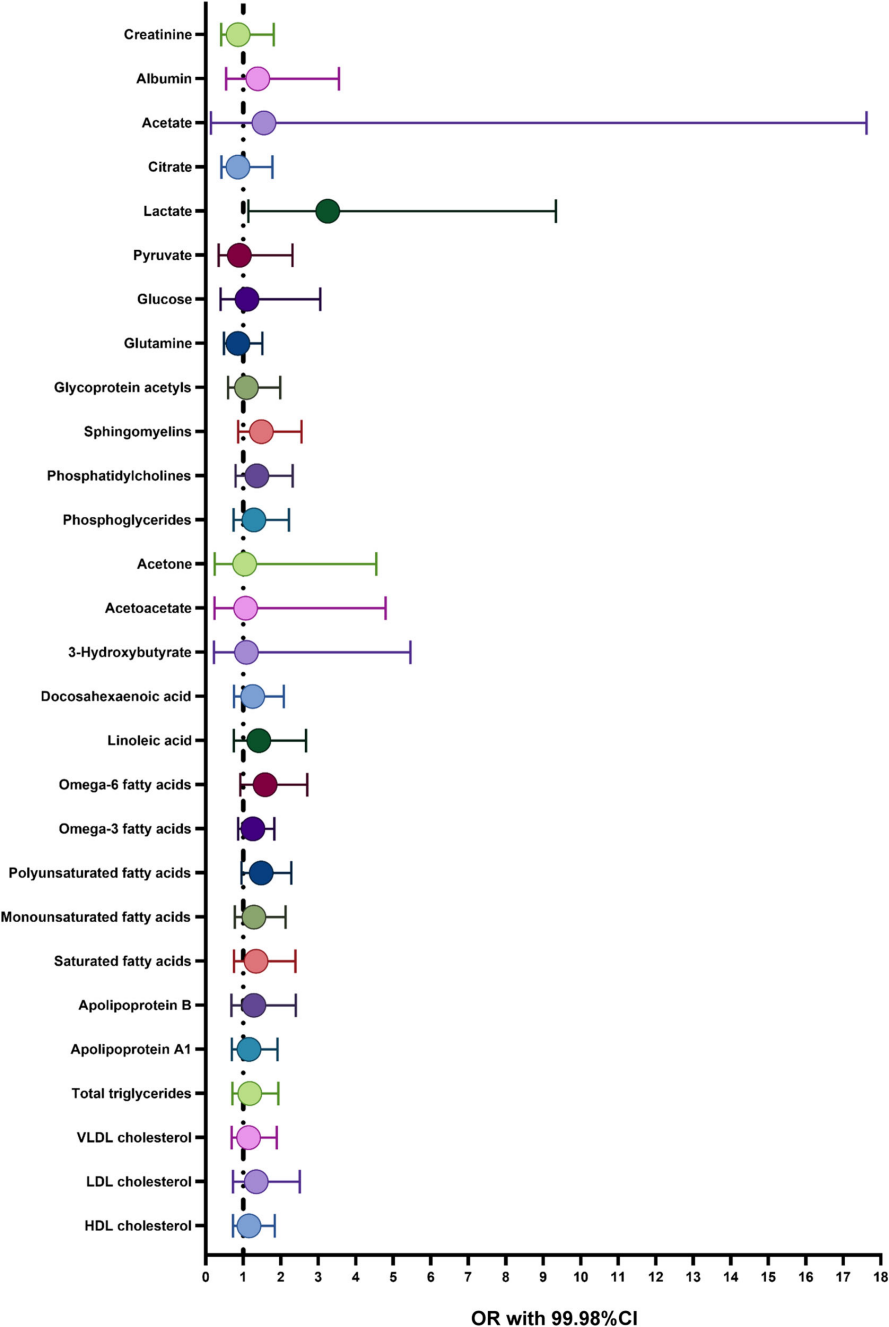
The causal effect of 28 common metabolites on kidney tumorigenesis based on Bonferrion-corrected *P* value (*P* < 2e–04). Error bars were defined as OR with 95% CI. VLDL very low density lipoprotein, LDL low density lipoprotein, HDL high density lipoprotein, OR odds ratio, CI confidence interval.

To set *P* value at a strict Bonferrion-corrected level (*P* < 2e–04), we only found two metabolites that were causally associated with renal cancer, among all the 249 metabolic biomarkers (Table 1). They were lactate (OR:3.25, 95% CI: 1.84-5.76, 99.98% CI: 1.13–9.33, *P* = 5.08e–05, Fig. 2 and Fig. S1) and phospholipids to total lipids ratio in large LDL (low density lipoprotein) (OR: 0.63, 95% CI: 0.50–0.80, 99.98% CI: 0.41–0.98, *P* = 1.39e-04, Fig. S2 and Fig. S3). For further details, common metabolites’ effect on renal cancer with 99.98% CI (Bonferrion-corrected *P* value) and 95% CI was illustrated in Fig. 2 and Fig. S1, respectively. While for phospholipid-related metabolites, two-sample MR effect was elucidated in Fig. S2 and S3. Details about each metabolite’s effect on kidney cancer and corresponding heterogeneity and pleiotropy test results were shown in Supplementary Data 2 (MR analysis results), Supplementary Data 3 (heterogeneity test results) and Supplementary Data 4 (pleiotropy test results).

**Table 1:** Details of the two significant traits after multiple two-sample MR tests with Bonferrion-corrected *P* value (*P* < 2e–04)

Exposure	Outcome	SNPs	Method	Beta	Se	*P* value	P_Heterogeneity_	P_Pleiotropy_
Phospholipids to total lipids ratio in large LDL	Kidney cancer	43	Random-effect IVW	−0.4587	0.1204	1.3942e-04		
			MR-Egger (bootstrap)	−0.4116	0.1718	5.0000e-03		
			IVW	−0.4587	0.1290	3.7817e-04	0.7073	
			MR-Egger	−0.3261	0.2077	1.2407e-01	0.6957	0.4198
			Weighted median	−0.4866	0.1856	8.7579e-03		
			MR-PRESSO	−0.4663	0.1159	2.1803e-04		0.8152
Lactate	Kidney cancer	12	Random-effect IVW	1.1798	0.2912	5.0759e-05		
			MR-Egger (bootstrap)	1.0318	1.1788	1.9300e-01		
			IVW	1.1798	0.3883	2.3767e-03	0.8607	
			MR-Egger	1.2362	1.2267	3.3736e-01	0.7996	0.9623
			Weighted median	1.0720	0.4988	3.1605e-02		
			MR-PRESSO	1.1798	0.2912	1.9087e-03		0.8860

*MR* Mendelian randomization; *SNPs* single nucleotide polymorphisms; *Se* standard error; *IVW* inverse variance weighted; *MR-PRESSO* Mendelian Randomization Pleiotropy RESidual Sum and Outlier.

### Further sensitivity analysis of significant metabolic biomarkers

**Fig. 3:**
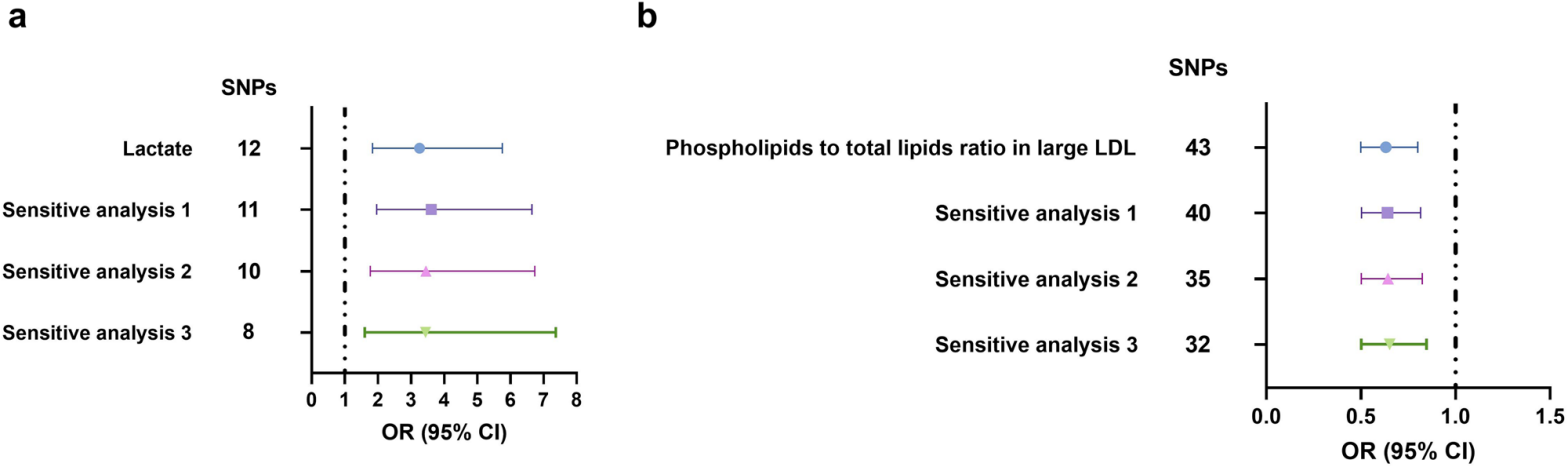
Sensitivity analysis of the significant metabolites filtered from all the 249 metabolites. **a** Sensitivity analysis of lactate effect on kidney cancer phenotype. **b** Sensitivity analysis of phospholipids to total lipids ratio in large LDL effect on kidney cancer phenotype. Error bars were defined as OR with 95% CI. SNPs single nucleotide polymorphisms, LDL low density lipoprotein, OR odds ratio, CI confidence interval.

After multiple two-sample MR analysis between 249 metabolic biomarkers and kidney cancer, two traits were considered significant (lactate and phospholipids to total lipids ratio in large LDL). The Steiger test results indicated a correct causal direction from the exposure variable (lactate and phospholipids to total lipids ratio in large LDL) to the outcome variable (kidney cancer, Supplementary Data 5). Then we applied detailed sensitivity analysis. First we removed SNPs that have been reported to be significantly correlated (*P* < 5e–08) with any cancer phenotype (defined as sensitivity analysis 1, Fig. 3a, b). Second, we removed SNPs significantly associated with blood pressure-related phenotypes on the basis of sensitivity analysis 1 (defined as sensitivity analysis 2, Fig. 3a, b). Third, we further conducted trait-specific sensitivity analysis (defined as sensitivity analysis 3, Fig. 3a, b). For lactate biomarker, which was thought to be involved in glycolysis, we removed SNPs significantly associated with lipid-related phenotypes on the basis of sensitivity analysis 2. While for the biomarker of phospholipids to total lipids ratio in large LDL, which was related to lipid metabolism, we removed SNPs significantly associated with glycemic phenotypes on the basis of sensitivity analysis 2. We described those SNPs that were utilized for sensitivity MR analysis in detail in Supplementary Data 5. Some SNPs might be left out due to their unavailability in the summary statistics of kidney cancer phenotype.

In our sensitivity analysis, we found all the results were stable (Fig. 3a, b). To be more specific, lactate was positively associated with kidney cancer, while phospholipids to total lipids ratio in large LDL was negatively correlated with kidney cancer (Fig. 3a, b). We further performed the reverse MR analysis to validate the positive results and avoid bidirectional causality. As a genome-wide *P* value threshold (5e–08) excluded all the SNPs of kidney cancer variable, we used a loose P value threshold (1e–05). The summary statistics of included SNPs were detailed in Supplementary Data 6a and Supplementary Data 6e. We did not find any causal effect of kidney cancer on lactate or phospholipids to total lipids ratio in large LDL (beta of random-effect IVW: 0.007 for lactate and -0.002 for phospholipids to total lipids ratio in large LDL, standard error: 0.004 for lactate and 0.004 for phospholipids to total lipids ratio in large LDL, P value: 0.138 for lactate and 0.602 for phospholipids to total lipids ratio in large LDL, Supplementary Data 6b and Supplementary Data 6f). The heterogeneity and pleiotropy effects were not observed (Supplementary Data 6c, Supplementary Data 6d, Supplementary Data 6g and Supplementary Data 6h).

## Discussion

In this study, we conducted multiple two-sample MR analysis to investigate the causal effect of 249 metabolic biomarkers on kidney cancer risk. We did find several metabolites had suggestive causal association with kidney cancer, which included lactate, sphingomyelins, phosphatidylcholines, linoleic acid, omega-6 fatty acids, omega-3 fatty acids and polyunsaturated fatty acids (Fig. S1). They were common products or components necessary for renal tumor development and growth. However, after we applied a more restricted P threshold, only two traits were thought to be significant. They were lactate (OR:3.25, 95% CI: 1.84–5.76, 99.98% CI: 1.13–9.33, *P* = 5.08e–05, Fig. 2 and Fig. S1) and phospholipids to total lipids ratio in large LDL (OR: 0.63, 95% CI: 0.50–0.80, 99.98% CI: 0.41–0.98, *P* = 1.39e–04, Fig. S2 and Fig. S3). These two metabolic traits played different roles in kidney tumorigenesis and the results were still stable through all the sensitivity analysis (Fig. 3a, b).

We stressed the influence of lactate in kidney tumorigenesis among all those metabolites. Actually, a recent publication revealed that perinephric adipose tissue could release excess lactate, which promoted clear cell RCC growth, invasion and metastasis^7^. The study reminded us that RCC was much involved with metabolism and lactate might serve as a key factor to facilitate the growth of kidney cancer. However, the evidence based on population of large sample size was lacking. On the other hand, numerous researches also discovered aberrant metabolites deposition in the tissue of renal cancer or in urine^4,11–18^. Nevertheless, all these were just correlation analysis and a causal effect could not be determined. Moreover, since RCC interacted with quantities of metabolites, it was difficult for us to figure out which ones were of great importance in kidney tumorigenesis according to conventional basic and clinical studies. Luckily, recent high-quality GWAS with large sample size gave us the opportunity to investigate the causal relationship between hundreds of metabolites and kidney cancer utilizing genetic instruments. Based on a strict P value threshold, we finally considered lactate was the most important pro-tumor factor in the development of kidney cancer. The results could guide the direction of future researches.

In addition to lactate, we also observed another metabolic biomarker, phospholipids to total lipids ratio in large LDL, was causally negatively associated with kidney cancer and might act as a protective factor. This was a novel insight. We could see from Fig. S3, higher phospholipids to total lipids ratio in both large and medium LDL reduced the risk of kidney cancer. How phospholipids interacted with renal cancer and whether the biomarker could be targeted as new therapy needed further investigations. However, it has been recognized that the peroxidation of phospholipids could trigger ferroptosis, a form of programmed cell death^19,20^. Recent studies illustrated that ferroptosis exerted anti-tumor effect on renal cancer^21–24^. From this point of view, we hypothesized that elevated phospholipids level posed a negative impact on kidney cancer through regulating ferroptosis. Our results provided a new basis for further investigations to focus on the effect of phospholipids on kidney cancer risk.

We found fatty acid might participate in kidney tumorigenesis, although the results did not pass the strict P value threshold. In our study, we tested the effect of docosahexaenoic acid, linoleic acid, omega-3 fatty acids, omega-6 fatty acids, polyunsaturated fatty acids, monounsaturated fatty acids and saturated fatty acids on kidney cancer. We just observed linoleic acid, omega-3 fatty acids, omega-6 fatty acids and polyunsaturated fatty acids had suggestive causal associations. These all belonged to unsaturated fatty acids, which served as necessary components of cell membrane to maintain its fluidity. We considered it was polyunsaturated fatty acids instead of monounsaturated fatty acids or saturated fatty acids that contributed to the growth of kidney cancer. Among those unsaturated fatty acids, omega-6 fatty acids held the strongest effect (OR: 1.58, 95% CI: 1.18-2.12). Actually, there had been one study exploring the role of fatty acid in renal tumor growth, despite the research did not focus on the exact type of fatty acid^8^. The results might inspire our thinking about the causal effect of unsaturated fatty acids, particularly the omega-6 fatty acids, on renal cancer development.

The study had several strengths. Firstly, we explored the causal relationship between 249 metabolic biomarkers and kidney cancer. We figured out that lactate was the most critical metabolite that impacted the growth of renal cancer, among all those metabolites. Secondly, we discovered the metabolic biomarker, phospholipids to total lipids ratio in large LDL, was negatively causally associated with kidney tumorigenesis. Such findings were interesting and could facilitate further investigations. Thirdly, we posed our reflection that polyunsaturated fatty acids rather than monounsaturated fatty acids or saturated fatty acids had the potential to induce RCC. Among those polyunsaturated fatty acids, omega-6 fatty acids attracted the most attention due to its suggestive effect size, although not significant.

The study also had limitations. Albeit we enrolled 249 metabolic biomarkers in our analysis, which covered most common metabolites, there were still several biomarkers not available in our study, for instance betaine and acetylcholine, as a result of lacking in powerful GWAS. Moreover, we performed sensitivity analysis to excluded SNPs associated with potential confounders as possible as we could. There also existed several unknown confounders that we might not exclude, which could violate the independence assumption. But substantially large sample size was included in our study, with strict Bonferrion-corrected P threshold for multiple tests and several sensitivity analysis. We could still draw our conclusions with sufficient power. In addition, it was frustrating that FinnGen Biobank did not provide summary statistics of kidney cancer with different clinical stages and histologies. That prevented us from exploring the causal effect of metabolites on the prognosis of kidney cancer. Moreover, the Finnish population is considered a bottleneck population and genetically differentiated from other European populations, so our results should be interpreted cautiously from this point if view.

In conclusion, we identified lactate, among all 249 metabolites, that was causally associated with kidney cancer growth. Moreover, we discovered the metabolic biomarker, phospholipids to total lipids ratio in large LDL, might serve as a protector to reduce kidney cancer risk. The results emphasized the central role of lactate in kidney tumorigenesis and provided novel insights into possible mechanism how phospholipids could affect kidney tumorigenesis. We were looking forward to subsequent experimental verification emphasizing on the causal role of phospholipids in kidney cancer risk.

## Methods

This study extracted data from previous genome wide association studies (GWAS) with large sample size. All the data were manually curated by the MRC Integrative Epidemiology Unit (IEU) at the University of Bristol, which could be accessed through the IEU OpenGWAS project^25,26^. Only European population were included.

### Metabolic biomarkers phenotype

The summary statistics data of metabolic biomarkers were derived from UK Biobank consortium, measured by Nightingale Health 2020 (https://www.ukbiobank.ac.uk/learn-more-about-uk-biobank/news/nightingale-health-and-uk-biobank-announces-major-initiative-to-analyse-half-a-million-blood-samples-to-facilitate-global-medical-research). Nightingale Health is a health technology company that provides a blood analysis platform for population-scale research and personalized health services. Its technology for profiling biomarkers was utilized to examine blood samples from the UK Biobank, measuring metabolic biomarkers that have been identified in recent studies as predictors of future risk for several common chronic diseases. In total, a variety of 249 metabolites was measured in blood samples from hundreds of thousands of participants (100,000 ~ ) and analyzed by tens of millions of single nucleotide polymorphisms (SNPs, 10,000,000 ~ ). Briefly, these metabolites contained glycemic, lipidic, amino acid-related, fatty acid-related and some other biomarkers. More details were described in Supplementary Data 1. The summary statistics data mainly included SNPs information (chromosome, position, effect allele, reference allele and the frequency of effect allele), their effect size on the concentration of each metabolite (beta, standard error and P value) and sample size information.

### Kidney cancer phenotype

We utilized kidney cancer data from FinnGen Biobank with 971 cases and 217,821 controls (Supplementary Data 1). The data excluded malignant neoplasm of renal pelvic, so only RCC samples were included. Generally, FinnGen is a large-scale academic-industry research consortium that aims to study the genetic and environmental factors underlying common chronic diseases in the Finnish population. FinnGen has established a biobank that includes genetic data and longitudinal health records from over 500,000 participants, making it one of the largest biobanks in the world. Its biobank contains comprehensive health data, including electronic health records, national health registers, and biobank samples from study participants. The data is collected from various sources, such as hospitals, health centers, and registries, and is linked with genetic data obtained from biobank samples. This allows for the identification of genetic and environmental factors that contribute to the development of common chronic diseases, including kidney cancer.

### Statistics and reproducibility

We conducted such an MR study to test the causal role of “exposure” (249 metabolites) in “outcome” (kidney cancer). First, significant SNPs (*P* < 5e–08) associated with each metabolic biomarker were extracted, after linkage disequilibrium (LD) clump (r^2^ < 0.001, kb = 10000). Then we extracted corresponding SNPs’ data from kidney cancer phenotype. During the process, proxy was allowed with minimum LD r^2^ equal to 0.8. In this way, the exposure and outcome data were harmonized and two-sample MR effect was calculated. The primary endpoint was MR causal effect calculated by random-effect inverse variance weighted (IVW) method. We also applied the other five methods for sensitivity analysis: IVW, MR Egger (bootstrap), MR Egger, weighted median and Mendelian Randomization Pleiotropy RESidual Sum and Outlier (MR-PRESSO) (Fig. 1). Heterogeneity and pleiotropy test were used. After completing 249 times of two-sample MR analysis, those metabolites with significant primary endpoints were included for further sensitivity analysis. In this way, we were able to investigate the causal effect of certain metabolites on kidney cancer.

All the analysis was based on R software (4.1.2). TwoSampleMR and ieugwasr were the main packages. The r square value was calculated as:

$${R}^{2}=2 * (1-MAF) * MAF * \frac{\beta }{{{{{{\rm{S}}}}}}E * \sqrt{N}}$$

In the equation, MAF and N referred to the minor allele frequencies and sample size, while β and SE referred to the effect size and standard error of the SNP. In our first step of multiple two-sample MR analysis, Bonferrion-corrected two-side P value was applied (P threshold equal to 0.05/249 = 2e–04) as a result of 249 tests (Fig. 1). The results were plotted as odds ratio (OR) with 99.98% confidence interval (CI), which was equivalent to the Bonferroni-corrected type I error rate alpha=2e-04. Any metabolite passed the threshold Bonferrion-corrected *P* value was considered significant. While metabolites with two-side *P* < 0.05 were thought to be suggestive. As we intended to investigate the causal effect of 249 metabolic biomarkers on kidney cancer, it was hard to remove all the SNPs significantly associated with confounders for each metabolites. Thus, we tried to include all the possible significant metabolites in our initial analysis. Metabolites with significant primary endpoints were included for further strict and comprehensive sensitivity analysis to validate the causal role of significant metabolites in kidney cancer risk. The sensitivity analysis included three steps:

i. Removal of SNPs significantly associated with any cancer phenotype, so as to eliminate possible pleiotropy;
ii. Removal of SNPs significantly associated with any cancer phenotype plus blood pressure-related phenotypes; so as to eliminate possible pleiotropy and confounders;
iii. Trait-specific sensitivity analysis according to the actual situation.

For such sensitivity analysis, OR with 95% CI was reported to validate previous results (Fig. 1).

In MR analysis, assumptions of instrumental variables were of great importance: relevance, independence and exclusion restriction. First, we extracted significant SNPs (*P* < 5e–08) associated with each metabolic biomarker to obey the relevance assumption. Then, sensitivity analysis to remove SNPs associated with any cancer phenotype (particularly kidney cancer) and potential confounders was applied to stick to the independence and exclusion restriction assumption. In addition, Steiger analysis was also performed to certify the direction of causality from the exposure variable to the outcome variable. Lastly, the reverse MR analysis was conducted to validate the positive results and avoid bidirectional causality.

### Reporting summary

Further information on research design is available in the Nature Portfolio Reporting Summary linked to this article.

### Supplementary information

Supplementary Information

Description of Additional Supplementary Materials

Supplementary Data 1

Supplementary Data 2

Supplementary Data 3

Supplementary Data 4

Supplementary Data 5

Supplementary Data 6

Reporting Summary

## Data Availability

All data generated or analyzed during this study are included in this published article and its supplementary information files (Supplementary Data 1–6). All the phenotypes could be accessed through the corresponding id number in Supplementary Data 1 from the IEU OpenGWAS project (https://gwas.mrcieu.ac.uk/).

## Code availability

All the analysis was based on R software (4.1.2). TwoSampleMR and ieugwasr were the main packages.
