# Supplementary material for: Investigating the causal associations between metabolic biomarkers and the risk of kidney cancer
Source: Commun Biol. 2024 Apr 1;7:398. doi: 10.1038/s42003-024-06114-8 (PMC10984917; doi:10.1038/s42003-024-06114-8)
Supplement: Supplementary file 1 — Supplementary Information [file 42003_2024_6114_MOESM1_ESM.pdf]

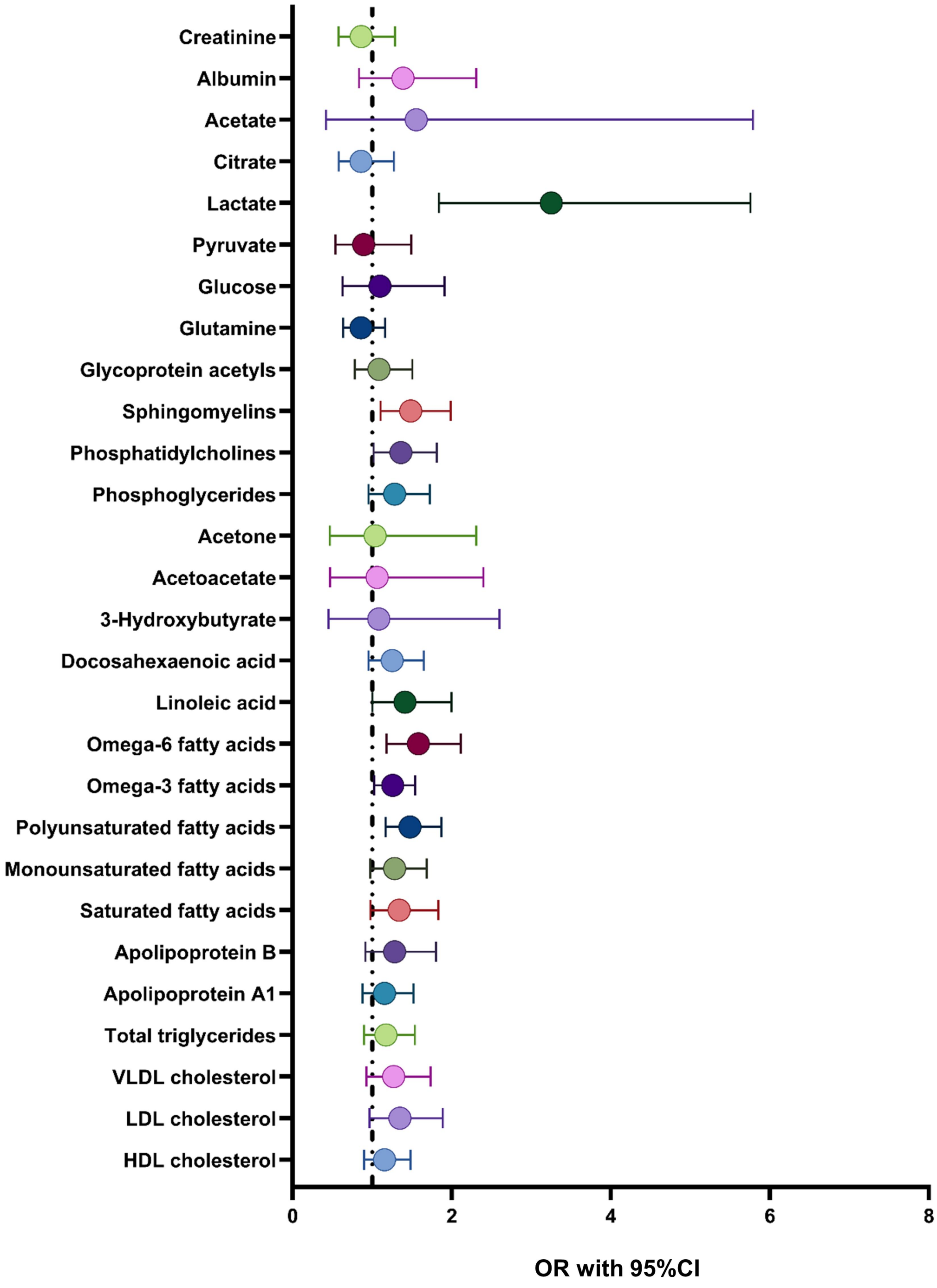

Figure S1. The causal effect of 28 common metabolites on kidney tumorigenesis based on suggestive P value (P<0.05).

Error bars were defined as OR with 95% CI.

VLDL: very low density lipoprotein; LDL: low density lipoprotein; HDL: high density lipoprotein; OR: odds ratio; CI: confidence interval.

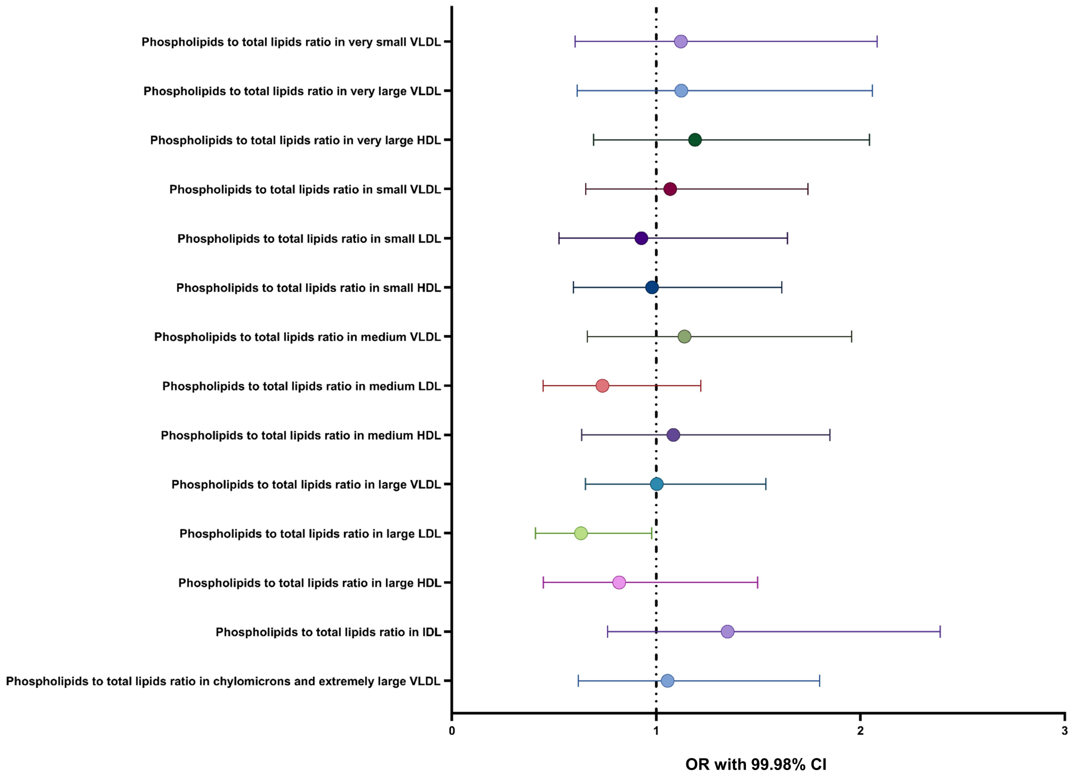

Figure S2. The causal effect of 14 phospholipid-related metabolites on kidney tumorigenesis based on Bonferrion-corrected P value (P<2e-04).

Error bars were defined as OR with 95% CI.

VLDL: very low density lipoprotein; LDL: low density lipoprotein; HDL: high density lipoprotein; OR: odds ratio; CI: confidence interval.

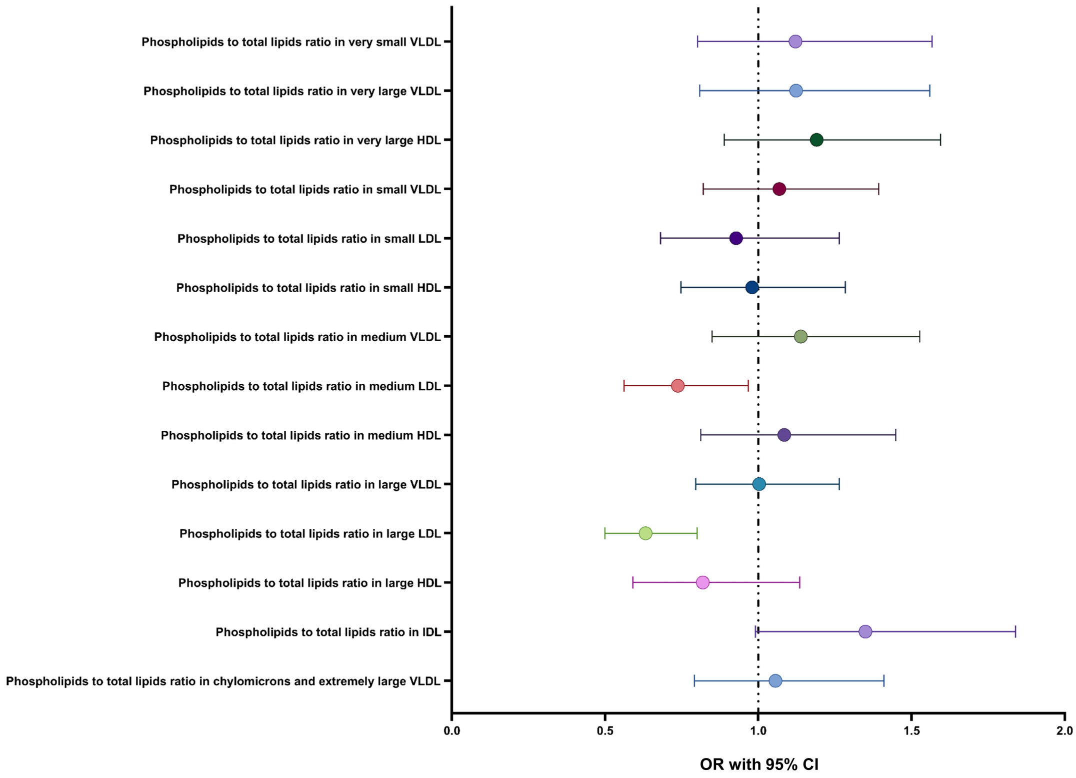

Figure S3. The causal effect of 14 phospholipid-related metabolites on kidney tumorigenesis based on suggestive P value (P<0.05).

Error bars were defined as OR with 95% CI.

VLDL: very low density lipoprotein; LDL: low density lipoprotein; HDL: high density lipoprotein; OR: odds ratio; CI: confidence interval.
