# Supplementary material for: Investigating the causal associations between metabolic biomarkers and the risk of kidney cancer
Source: Commun Biol. 2024 Apr 1;7:398. doi: 10.1038/s42003-024-06114-8 (PMC10984917; doi:10.1038/s42003-024-06114-8)
Supplement: Supplementary file 2 — Description of Additional Supplementary Materials [file 42003_2024_6114_MOESM2_ESM.docx]

**Description of Additional Supplementary Files**

**File name:** Supplementary Data 1

**Description:** Supplementary Data 1a. Phenotypes included in this study. Supplementary Data 1b. Detailed metabolites included in this study.

**File name:** Supplementary Data 2

**Description:** Mendelian randomization results of the causal effects of 249 metabolites on kidney cancer outcome.

**File name:** Supplementary Data 3

**Description:** Heterogeneity results of the causal effects of 249 metabolites on kidney cancer outcome.

**File name:** Supplementary Data 4

**Description:** Pleiotropy results of the causal effects of 249 metabolites on kidney cancer outcome.

**File name:** Supplementary Data 5

**Description:** SNP summary and sensitivity analysis results of the two metabolites.

**File name:** Supplementary Data 6

**Description:** Supplementary Data 6a. The summary statistics of included SNPs for the reverse MR analysis between kidney cancer and lactate. Supplementary Data 6b. The MR results of the effect of kidney cancer on lactate. Supplementary Data 6c. The heterogeneity results of the effect of kidney cancer on lactate. Supplementary Data 6d. The pleiotropy results of the effect of kidney cancer on lactate. Supplementary Data 6e. The summary statistics of included SNPs for the reverse MR analysis between kidney cancer and phospholipids to total lipids ratio in large LDL. Supplementary Data 6f. The MR results of the effect of kidney cancer on phospholipids to total lipids ratio in large LDL. Supplementary Data 6g. The heterogeneity results of the effect of kidney cancer on phospholipids to total lipids ratio in large LDL. Supplementary Data 6h. The pleiotropy results of the effect of kidney cancer on phospholipids to total lipids ratio in large LDL.
