# Supplementary material for: Investigating the causal associations between metabolic biomarkers and the risk of kidney cancer
Source: Commun Biol. 2024 Apr 1;7:398. doi: 10.1038/s42003-024-06114-8 (PMC10984917; doi:10.1038/s42003-024-06114-8)
Supplement: Supplementary file 9 — Reporting Summary [file 42003_2024_6114_MOESM9_ESM.pdf]

nature portfolio

Corresponding author(s): Xiang Li, Xu Hu

Last updated by author(s): Mar 23, 2024

Reporting Summary

Nature Portfolio wishes to improve the reproducibility of the work that we publish. This form provides structure for consistency and transparency in reporting. For further information on Nature Portfolio policies, see our Editorial Policies and the Editorial Policy Checklist.

Statistics

For all statistical analyses, confirm that the following items are present in the figure legend, table legend, main text, or Methods section.

n/a	Confirmed	
☐	☒	The exact sample size (*n*) for each experimental group/condition, given as a discrete number and unit of measurement
☐	☒	A statement on whether measurements were taken from distinct samples or whether the same sample was measured repeatedly
☐	☒	The statistical test(s) used AND whether they are one- or two-sided<br>*Only common tests should be described solely by name; describe more complex techniques in the Methods section.*
☐	☒	A description of all covariates tested
☐	☒	A description of any assumptions or corrections, such as tests of normality and adjustment for multiple comparisons
☐	☒	A full description of the statistical parameters including central tendency (e.g. means) or other basic estimates (e.g. regression coefficient) AND variation (e.g. standard deviation) or associated estimates of uncertainty (e.g. confidence intervals)
☐	☒	For null hypothesis testing, the test statistic (e.g. *F*, *t*, *r*) with confidence intervals, effect sizes, degrees of freedom and *P* value noted<br>*Give P values as exact values whenever suitable.*
☒	☐	For Bayesian analysis, information on the choice of priors and Markov chain Monte Carlo settings
☒	☐	For hierarchical and complex designs, identification of the appropriate level for tests and full reporting of outcomes
☐	☒	Estimates of effect sizes (e.g. Cohen's *d*, Pearson's *r*), indicating how they were calculated

Our web collection on statistics for biologists contains articles on many of the points above.

Software and code

Policy information about availability of computer code

Data collection

Metabolic biomarkers phenotype
The summary statistics data of metabolic biomarkers were derived from UK Biobank consortium, measured by Nightingale Health 2020 (https://www.ukbiobank.ac.uk/learn-more-about-uk-biobank/news/nightingale-health-and-uk-biobank-announces-major-initiative-to-analyse-half-a-million-blood-samples-to-facilitate-global-medical-research). Nightingale Health is a health technology company that provides a blood analysis platform for population-scale research and personalized health services. Its technology for profiling biomarkers was utilized to examine blood samples from the UK Biobank, measuring metabolic biomarkers that have been identified in recent studies as predictors of future risk for several common chronic diseases. In total, a variety of 249 metabolites was measured in blood samples from hundreds of thousands of participants (100,000~) and analyzed by tens of millions of single nucleotide polymorphisms (SNPs, 10,000,000~). Briefly, these metabolites contained glycemic, lipidic, amino acid-related, fatty acid-related and some other biomarkers. More details were described in Supplementary Data 1. The summary statistics data mainly included SNPs information (chromosome, position, effect allele, reference allele and the frequency of effect allele), their effect size on the concentration of each metabolite (beta, standard error and P value) and sample size information.

Kidney cancer phenotype
We utilized kidney cancer data from FinnGen Biobank with 971 cases and 217,821 controls (Supplementary Data 1). The data excluded malignant neoplasm of renal pelvic, so only RCC samples were included. Generally, FinnGen is a large-scale academic-industry research consortium that aims to study the genetic and environmental factors underlying common chronic diseases in the Finnish population. FinnGen has established a biobank that includes genetic data and longitudinal health records from over 500,000 participants, making it one of the largest biobanks in the world. Its biobank contains comprehensive health data, including electronic health records, national health registers, and biobank samples from study participants. The data is collected from various sources, such as hospitals, health centers, and registries, and

is linked with genetic data obtained from biobank samples. This allows for the identification of genetic and environmental factors that contribute to the development of common chronic diseases, including kidney cancer.

Data analysis

Statistics and reproducibility
We conducted such an MR study to test the causal role of "exposure" (249 metabolites) in "outcome" (kidney cancer). First, significant SNPs (P <5e-08) associated with each metabolic biomarker were extracted, after linkage disequilibrium (LD) clump (r2 <0.001, kb=10000). Then we extracted corresponding SNPs' data from kidney cancer phenotype. During the process, proxy was allowed with minimum LD r2 equal to 0.8. In this way, the exposure and outcome data were harmonized and two-sample MR effect was calculated. The primary endpoint was MR causal effect calculated by random-effect inverse variance weighted (IVW) method. We also applied the other five methods for sensitivity analysis: IVW, MR Egger (bootstrap), MR Egger, weighted median and Mendelian Randomization Pleiotropy RESidual Sum and Outlier (MR-PRESSO) (Figure 1). Heterogeneity and pleiotropy test were used. After completing 249 times of two-sample MR analysis, those metabolites with significant primary endpoints were included for further sensitivity analysis. In this way, we were able to investigate the causal effect of certain metabolites on kidney cancer.

All the analysis was based on R software (4.1.2). TwoSampleMR and ieugwasr were the main packages. The r square value was calculated as:
.
In the equation, MAF and N referred to the minor allele frequencies and sample size, while β and SE referred to the effect size and standard error of the SNP. In our first step of multiple two-sample MR analysis, Bonferrion-corrected two-side P value was applied (P threshold equal to 0.05/249=2e-04) as a result of 249 tests (Figure 1). The results were plotted as odds ratio (OR) with 99.98% confidence interval (CI), which was equivalent to the Bonferroni-corrected type I error rate alpha=2e-04. Any metabolite passed the threshold Bonferrion-corrected P value was considered significant. While metabolites with two-side P<0.05 were thought to be suggestive. As we intended to investigate the causal effect of 249 metabolic biomarkers on kidney cancer, it was hard to remove all the SNPs significantly associated with confounders for each metabolites. Thus, we tried to include all the possible significant metabolites in our initial analysis. Metabolites with significant primary endpoints were included for further strict and comprehensive sensitivity analysis to validate the causal role of significant metabolites in kidney cancer risk. The sensitivity analysis included three steps:
i.Removal of SNPs significantly associated with any cancer phenotype, so as to eliminate possible pleiotropy;
ii.Removal of SNPs significantly associated with any cancer phenotype plus blood pressure-related phenotypes; so as to eliminate possible pleiotropy and confounders;
iii.Trait-specific sensitivity analysis according to the actual situation.
For such sensitivity analysis, OR with 95% CI was reported to validate previous results (Figure 1).

In MR analysis, assumptions of instrumental variables were of great importance: relevance, independence and exclusion restriction. First, we extracted significant SNPs (P <5e-08) associated with each metabolic biomarker to obey the relevance assumption. Then, sensitivity analysis to remove SNPs associated with any cancer phenotype (particularly kidney cancer) and potential confounders was applied to stick to the independence and exclusion restriction assumption. In addition, Steiger analysis was also performed to certify the direction of causality from the exposure variable to the outcome variable. Lastly, the reverse MR analysis was conducted to validate the positive results and avoid bidirectional causality.

For manuscripts utilizing custom algorithms or software that are central to the research but not yet described in published literature, software must be made available to editors and reviewers. We strongly encourage code deposition in a community repository (e.g. GitHub). See the Nature Portfolio guidelines for submitting code & software for further information.

Data

Policy information about availability of data

All manuscripts must include a data availability statement. This statement should provide the following information, where applicable:

- Accession codes, unique identifiers, or web links for publicly available datasets
- A description of any restrictions on data availability
- For clinical datasets or third party data, please ensure that the statement adheres to our policy

All data generated or analyzed during this study are included in this published article and its supplementary information files (Supplementary Data 1-6). All the phenotypes could be accessed through the corresponding id number in Supplementary Data 1 from the IEU OpenGWAS project (https://gwas.mrcieu.ac.uk/).

Research involving human participants, their data, or biological material

Policy information about studies with human participants or human data. See also policy information about sex, gender (identity/presentation), and sexual orientation and race, ethnicity and racism.

Reporting on sex and gender	Not applicable.
Reporting on race, ethnicity, or other socially relevant groupings	European population were included.
Population characteristics	Supplementary Data 1.
Recruitment	The study used publicly available datasets, details of which could be accessed through https://gwas.mrcieu.ac.uk/.
Ethics oversight	The study used publicly available datasets, details of which could be accessed through https://gwas.mrcieu.ac.uk/.

Note that full information on the approval of the study protocol must also be provided in the manuscript.

Field-specific reporting

Please select the one below that is the best fit for your research. If you are not sure, read the appropriate sections before making your selection.

☒ Life sciences ☐ Behavioural & social sciences ☐ Ecological, evolutionary & environmental sciences

For a reference copy of the document with all sections, see nature.com/documents/nr-reporting-summary-flat.pdf

Life sciences study design

All studies must disclose on these points even when the disclosure is negative.

Sample size	Hundreds of thousands of participants were included in Supplementary Data 1. The study used publicly available datasets, details of which could be accessed through https://gwas.mrcieu.ac.uk/.
Data exclusions	First, significant SNPs (P <5e-08) associated with each metabolic biomarker were extracted, after linkage disequilibrium (LD) clump (r2 <0.001, kb=10000). Then we extracted corresponding SNPs' data from kidney cancer phenotype. During the process, proxy was allowed with minimum LD r2 equal to 0.8. In this way, the exposure and outcome data were harmonized and two-sample MR effect was calculated.
Replication	The primary endpoint was MR causal effect calculated by random-effect inverse variance weighted (IVW) method. We also applied the other five methods for sensitivity analysis: IVW, MR Egger (bootstrap), MR Egger, weighted median and Mendelian Randomization Pleiotropy RESidual Sum and Outlier (MR-PRESSO) (Figure 1). Heterogeneity and pleiotropy test were used. After completing 249 times of two-sample MR analysis, those metabolites with significant primary endpoints were included for further sensitivity analysis. In addition, Steiger analysis was also performed to certify the direction of causality from the exposure variable to the outcome variable. Lastly, the reverse MR analysis was conducted to validate the positive results and avoid bidirectional causality.
Randomization	Not applicable.
Blinding	Not applicable.

Reporting for specific materials, systems and methods

We require information from authors about some types of materials, experimental systems and methods used in many studies. Here, indicate whether each material, system or method listed is relevant to your study. If you are not sure if a list item applies to your research, read the appropriate section before selecting a response.

Materials & experimental systems

n/a	Involved in the study
☒	☐ Antibodies
☒	☐ Eukaryotic cell lines
☒	☐ Palaeontology and archaeology
☒	☐ Animals and other organisms
☒	☐ Clinical data
☒	☐ Dual use research of concern
☒	☐ Plants

Methods

n/a	Involved in the study
☒	☐ ChIP-seq
☒	☐ Flow cytometry
☒	☐ MRI-based neuroimaging

Plants

Seed stocks	Not applicable.
Novel plant genotypes	Not applicable.
Authentication	Not applicable.
